# Accurate and fiducial-marker-free correction for three-dimensional chromatic shift in biological fluorescence microscopy

**DOI:** 10.1038/s41598-018-25922-7

**Published:** 2018-05-15

**Authors:** Atsushi Matsuda, Lothar Schermelleh, Yasuhiro Hirano, Tokuko Haraguchi, Yasushi Hiraoka

**Affiliations:** 1 Advanced ICT Research Institute Kobe, National Institute of Information and Communications Technology, 588-2 Iwaoka, Iwaoka-cho, Nishi-ku, Kobe, 651-2492 Japan; 2 Graduate School of Frontier Biosciences, Osaka University, 1-3 Yamadaoka, Suita, 565-0871 Japan; 3 Micron Advanced Bioimaging Unit, Department of Biochemistry, University of Oxford, South Parks Road, Oxford, OX1 3QU United Kingdom

## Abstract

Correction of chromatic shift is necessary for precise registration of multicolor fluorescence images of biological specimens. New emerging technologies in fluorescence microscopy with increasing spatial resolution and penetration depth have prompted the need for more accurate methods to correct chromatic aberration. However, the amount of chromatic shift of the region of interest in biological samples often deviates from the theoretical prediction because of unknown dispersion in the biological samples. To measure and correct chromatic shift in biological samples, we developed a quadrisection phase correlation approach to computationally calculate translation, rotation, and magnification from reference images. Furthermore, to account for local chromatic shifts, images are split into smaller elements, for which the phase correlation between channels is measured individually and corrected accordingly. We implemented this method in an easy-to-use open-source software package, called *Chromagnon*, that is able to correct shifts with a 3D accuracy of approximately 15 nm. Applying this software, we quantified the level of uncertainty in chromatic shift correction, depending on the imaging modality used, and for different existing calibration methods, along with the proposed one. Finally, we provide guidelines to choose the optimal chromatic shift registration method for any given situation.

## Introduction

Multicolor imaging is a key modality in biological fluorescence microscopy to determine the relationships between different targets within a specimen. Even though recent developments in super-resolution microscopy have improved spatial resolution to tens of nanometers^1–3^, accurate measurements of three-dimensional (3D) spatial relationships between two or more kinds of molecules using multicolor imaging still pose a significant challenge. Due to dispersion, i.e., the dependence of the refractive index on light’s wavelength, apparent fluorescence distributions are shifted in space and moved up or down if multicolor images are simply overlaid. Such chromatic aberration along the axial and lateral directions may lead to incorrect conclusions if not appropriately corrected. Furthermore, some high-end microscopes are equipped with multiple cameras for different channels, making multicolor images more difficult to overlay. As the resolution of fluorescence microscopy is increased, the precision of correction, or “registration”, for chromatic shift also needs to be increased. Various registration methods have been presented that can correct the chromatic shift at or around the surface of a coverslip^4–10^. These typically rely on fiducial markers analyzed separately from the sample of interest (e.g., a 2D layer of multispectral fluorescent beads attached to the coverslip). Registration parameters are then calculated from marker’s coordinates, and finally a transformation matrix is applied to the target’s multicolor images. Most biological imaging is, however, three-dimensional, with a depth of interest often tens of micrometers away from the coverslip surface. As Manders pointed out two decades ago^4^, chromatic shift depends on the embedding medium and the depth of focus. Therefore, for 3D multicolor imaging, chromatic shift has to be measured using the sample of interest at the depth of interest, but, to our knowledge, very few studies have addressed this problem. Some researchers carefully introduced fiducial markers inside the samples^7,11^, but such approaches are not always feasible for common biological samples.

To measure sample-induced chromatic shifts, two methods have been recently proposed: “biological calibration slide”^12^ and “cross-talk on demand”^13–15^. The biological calibration slide method uses a calibration slide of biological samples that is prepared in a similar way as the sample slides, but a suitable target is simultaneously stained by multiple color dyes. Because biological samples of the same kind prepared in the same way should have similar dispersion, the chromatic shift can be inferred from such a biological calibration slide. The other approach (cross-talk on demand) uses cellular features ‘mixed’ into the respective channels by fluorescence bleed-through as markers for alignment^13–15^. The cross-talk on demand approach uses point-like features. For example, images of nuclear pore complexes observed in the yellow and red channels by bleed-through of tdTomato fluorescent protein was used to align the two channels^13^. However, biological images do not always contain point-like objects.

In this work, we devised a new method to calculate registration parameters from any type of biological image, allowing for fluorescent-bead-free measurements of chromatic shift. We implemented this method in an easy-to-use stand-alone software, named “*Chromagnon*”. Using this software, we were able to examine how much uncertainty in registration accuracy was to be expected using preexisting methods. Additionally, we present a simple yet accurate method to correct chromatic shift.

## Results

### Correlation-based acquisition of the registration parameters for any biological image

**Figure 1:**
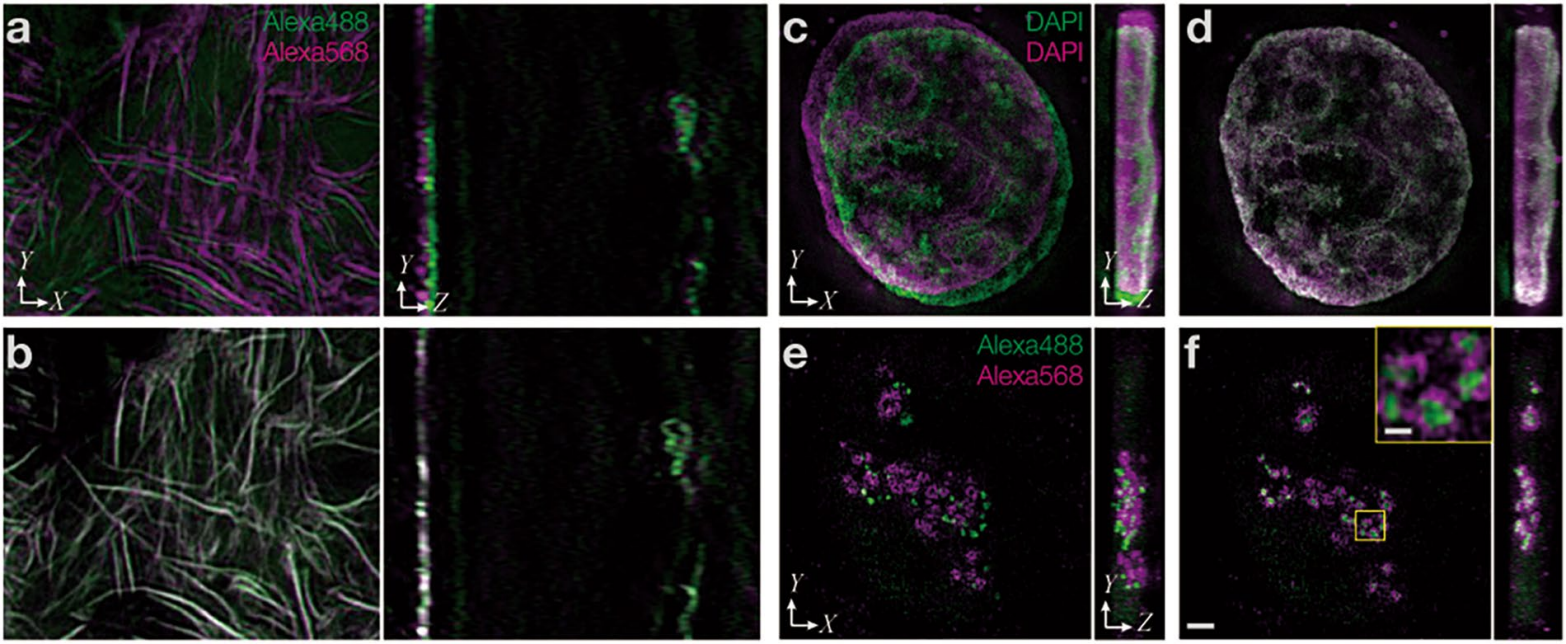
Registration methods to measure and correct chromatic shift using biological samples. Representative 3D-SIM images. (**a**) Actin filaments stained with dye-conjugated phalloidin. The same image is shown in the green (shown in green) and orange (shown in purple) emission ranges, detected simultaneously with different cameras. (**b**) The same image as in (**a**) after chromatic correction. (**c**) Nuclear DNA stained with DAPI excited at 405 nm. Images of normally discarded bleed-through signals in the green and orange emission ranges, detected simultaneously with different cameras. (**d**) The same image as in (**c**) after chromatic correction. (**e**) Nucleolar proteins, treacle and fibrillarin, stained with Alexa Fluor 488 and 568 in the same cell and shown at the same focus position as in (**c**). Images were acquired by sequential excitation with 488 and 561 nm, for detection in the green and orange emission channels, respectively. (**f**) The same image as in (**e**) after registration using the same parameters used to align (**c**). The boxed region was magnified and is shown in the inset. The scale bar is 2 µm for panels (**a**–**f**) and 0.5 µm for the inset in (**f**).

Both the biological calibration slide and cross-talk on demand approaches would benefit from being able to use any biological image as a marker for channel alignment. For the biological calibration slides method, a single primary antibody and multicolor secondary antibodies can co-stain a single target structure in multiple colors. Due to their higher target density, perhaps even better suited for multi-color co-staining are small molecule labeling reagents, such as phalloidin, concanavalin A, and 5-ethynyl-2′-deoxyuridine (EdU), detected by click chemistry as shown in Fig. 1a,b. Although the cross-talk on demand approach normally uses point-like structures, here we used the nucleus stained with 4′,6-diamidino-2-phenylindole (DAPI) as a cross-talk staining agent. DAPI has its emission peak at 461 nm, but shows emission in the green and orange channels as well. Thus, when exciting with a DAPI-specific wavelength (e.g., using a 405-nm laser line module), the emission signal of an object (in this case the cell nucleus) can be detected in all channels, albeit at decreasing intensities (Fig. 1c,d). Such images are suitable for the cross-talk approach.

**Figure 2:**
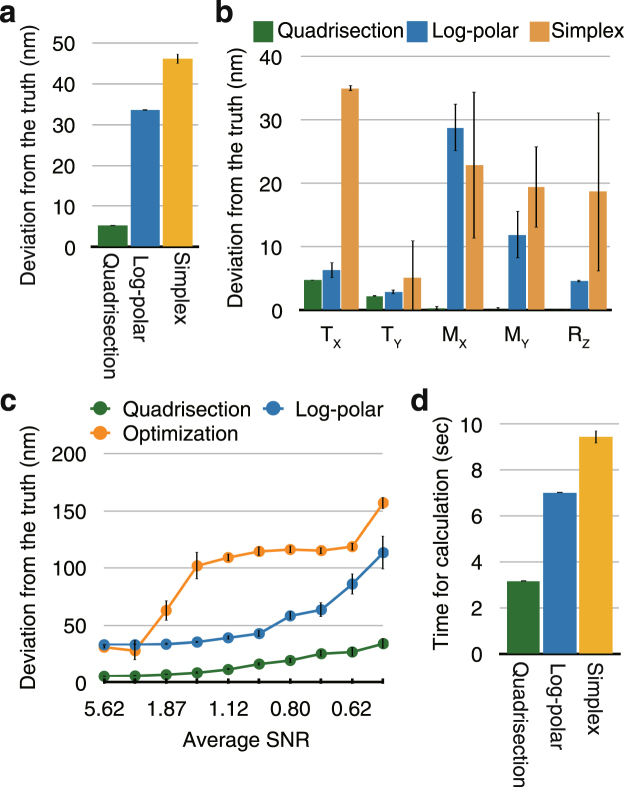
Comparison of the performances of the three calculation methods. (**a**) Comparison of registration performance using simulated data. An image stack of tubulin stained with CF405M was two-dimensionally shifted a known amount (see Methods or Supplementary Fig. S1a). Then, a maximum intensity projection was used to calculate the 2D registration error using each method. Deviation from the known registration parameters, i.e., the vector sum of the five parameters T_X_, T_Y_, M_X_, M_Y_, and R_Z_, is shown. The bars indicate the standard errors of twelve repetitions of the simulation. ‘Quadrisection’ stands for quadrisection phase correlation (see Results and Fig. 3); ‘Log-polar’ stands for log-polar transformation combined with phase correlation; and ‘Simplex’ stands for an optimization method using the simplex algorithm. (**b**) The same data in (**a**) shown for the individual parameters: translation along the X and Y axes (T_X_, T_Y_), magnification along the X and Y axes (M_X_, M_Y_), and rotation around the Z axis (R_Z_). (**c**) Comparison of noise-tolerance using simulated data. Both channels of the image created as explained in (**a**) were divided by constants ranging from 50 to 500, and either Gaussian or Poisson noise images with a standard deviation of 10 were added to both channels. The two datasets using Gaussian and Poisson noises were combined. Deviation from the known registration parameters is plotted as a function of average SNR (also see Supplementary Fig. S1). (**d**) Comparison of the time required for the calculations of 2D registration data of 508 × 508 pixels using eight cores of a Xeon E5-2623 v4 2.6 GHz processor. The bars indicate the standard error after 252 calculations.

We devised a workflow to measure the chromatic shifts throughout the volume of any kind of 3D image in a data set. While a number of methods have been proposed to calculate registration parameters from biological images^16^, we aimed to maximize accuracy for 3D registration parameters by calculating the translation (T_X_, T_Y_, and T_Z_) and magnification (M_X_, M_Y_, and M_Z_) in both the lateral (XY) and axial (Z) dimensions, as well as the rotation around the optical axis (R_Z_), and to implement this method in a versatile and robust software solution. Initially, we believed that log-polar transformation combined with cross-correlation or its derivative “phase-correlation” (hereafter referred to as the “log-polar method”^17^) and optimization-based methods (we used the simplex algorithm^18^) met our needs. However, these methods were found to have insufficient accuracy in our simulations (Fig. 2a,b). The reasons for the inaccuracy of the log-polar method are presumably the fact that the magnification factor cannot be separately measured for the X and Y axes and the insufficient resolution due to deformation caused by the log-polar transformation. The accuracy obtained using the simplex method was similar to that of the log-polar method (Fig. 2a,b), but the simplex method was far less robust than the log-polar method in the presence of simulated noise (Fig. 2c and Supplementary Fig. S1).

**Figure 3:**
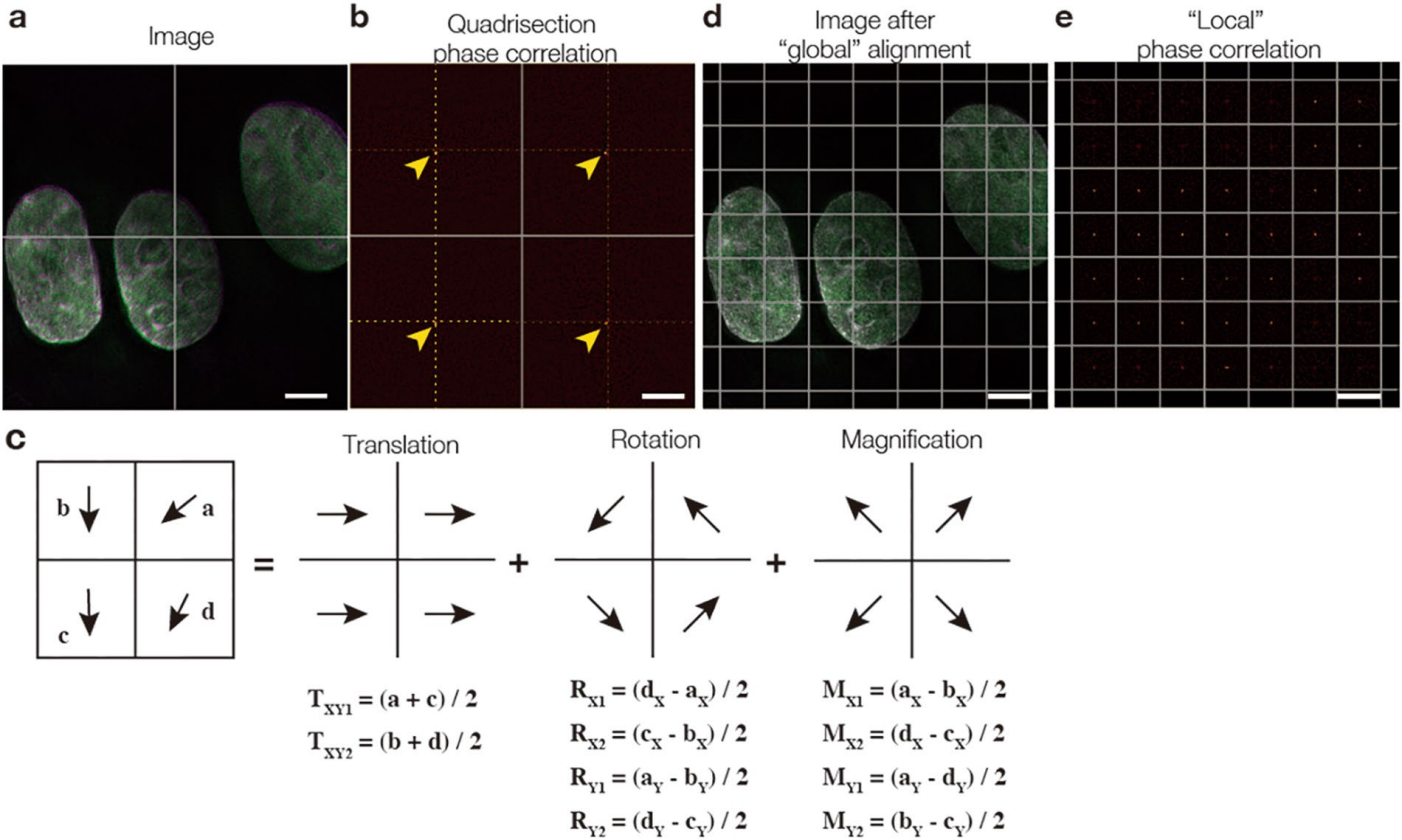
Principles of quadrisection phase correlation and local phase correlation. (**a**) A 3D-SIM image of DAPI-stained cell nuclei acquired in two channels, where a 2D section is split into four regions (shown as cross lines). For each quadrisection, the phase correlation between the two channels is measured. (**b**) The resulting image has four identifiable correlation peaks (arrowheads) around the center (cross-marked by dotted lines). The shifts of each individual peak from the center of the quadrisection indicates the direction and magnitude of the necessary shift to align the two channels in each individual quadrisection image. Such shifts are shown as vectors (**a**–**d**) in (**c**). If we assume that the shifts consist only of affine parameters (translation, magnification, and rotation), then the vectors should be the sums of the vectors corresponding to translation, rotation, and magnification. Therefore, adding vectors (**a** and **c**) extracts the translation part of the vectors because the rotation and magnification parts of the vectors should have an opposing orientation with equal length and thus should cancel each other out. This way, by cancelling out the other components (rotation and magnification), two independent translation vectors (**T**_**XY1**_ and **T**_**XY2**_) are obtained, and are then averaged to obtain the total translation **T**_**XY**_. Similar calculations yield the solutions for rotation and magnification. (**d**) A 2D section of the image shown in (**a**) after registration, where quadrisection phase correlation is split into windows of, for example, 128 × 128 pixels. For each window, the phase correlations between the two channels are measured, resulting in images as displayed in (**e**), where the shift of the individual peaks in each window indicate the direction and amount of shift necessary to align the two channels in each individual window. The local shifts were applied to the target images using a nonlinear elastic transformation. See Methods for more details. Scale bars are 5 µm.

For this reason, we devised a new calculation method, named “quadrisection phase correlation” (Fig. 3a–c), that can be used to precisely determine translation, magnification, and rotation. An image was split into four regions (Fig. 3a) and, for each region, phase correlations between two channels were obtained. The shifts of the four correlation peaks from the centers of the quadrisections (Fig. 3b) indicate the direction and the amount of the shift necessary to align two channels in individual quadrisection images, shown as vectors in Fig. 3c. Affine parameters (translation, magnification, and rotation) from quadrisection phase correlation were then obtained as a solution to the equations shown in Fig. 3c. For example, if we label the vectors from phase correlation of a quadrant as **a**, **b**, **c**, and **d** (Fig. 3c), and these vectors are the result of a sum of vectors coming from translation, magnification, and rotation (Fig. 3c), then adding **a** and **c** will double only the vector component of translation, but will cancel out the components of magnification and rotation (Fig. 3c). Consequently, (**a** + **c**)/2 extracts only the amount of translation (T_XY1_ in Fig. 3c) from the mixture of translation, magnification, and rotation. Similarly, (**b** + **d**)/2 also extracts the amount of translation (T_XY2_ in Fig. 3c). Thus, we determined T_XY_ by averaging T_XY1_ and T_XY2_. The vector components of rotation and magnification can also be extracted with similar operations, as shown in Fig. 3c. Although quadrisection phase correlation extracts global alignment parameters only from a 2D section, these calculations were performed for projections along the X or Z axes to measure the global 3D chromatic shift. Thus, seven registration parameters were obtained: translation along the X, Y, and Z axes; magnification along the X, Y, and Z axes; and rotation around the Z axis (i.e., around the optical axis; see Supplementary Table S1 for representative alignment parameters with our microscope).

The registration accuracy obtained through quadrisection phase correlation (~5 nm in 2D) was sufficient for the enhanced spatial resolution (15–150 nm) of super-resolution microscopy (Fig. 2a,b). In addition, this method surpassed existing methods in robustness (see Fig. 2c and Supplementary Fig. S1) and speed (shown in Fig. 2d). Furthermore, it was also largely unaffected by the presence of additional unrelated signals merging with the bleed-through signal of the reference structure; registration parameters deviated by ~30 nm, even though the signal intensity of the unrelated image was approximately the same as that of the true image (see Supplementary Fig. S2). The alignment performed with quadrisection phase correlation is hereafter referred to as global alignment. This method can be used to quantitatively measure and compare separable components (T_X_, T_Y_, T_Z_, M_X_, M_Y_, M_Z_, and R_Z_) of chromatic shift. The resulting registration parameters can be applied using a linear (rigid) transformation to target multicolor images that were acquired separately (Fig. 1b,d,f). This method is scale-free, that is, it can be applied to target images of any sizes.

Chromatic shift is not always uniform over the field of view, but locally distorted due to complex sample structures, irregularity of camera pixels, mirrors, or glasses. Therefore, after global alignment, we measured local chromatic shift by separating images into smaller pieces (Fig. 3d,e) by using a previously reported method^19^ with some modifications (see Methods for details). Briefly, we first split the images into 4 × 4 regions, measured the phase correlation between two channels, and obtained regional translation vectors for each of the 4 × 4 regions. The resulting registration parameters were then applied to the image using a nonlinear (elastic) transformation. The window size was then further halved and the same operation was repeated iteratively until the window size reached <60 pixels to obtain a fine map of regional translation vectors. The obtained local registration parameters were finally applied to the target image to correct for the differential local chromatic shift. Unlike the global alignment process, this method is not scale-free, that is, it cannot estimate chromatic shift beyond the reference image (example parameter outputs are shown in Supplementary Fig. S3).

The registration accuracy of the local alignment method depends on the signal to noise ratio (SNR) of the reference images and the minimum window size for phase correlation in our simulation (Supplementary Fig. S4). In general, when the minimum window size was smaller, the mean registration error was also smaller (<4 nm) at higher average SNRs (e.g., 14), but the error increased steeply at lower average SNRs (e.g., 2, see Supplementary Fig. S4c). With the window size of our default value of 60 pixels, the mean alignment errors in the map were in the 10.8–21.3 nm range at average SNRs of 1.88–2.15. Therefore, the local alignment method requires a higher SNR than the global alignment method (see Fig. 2c).

**Figure 4:**
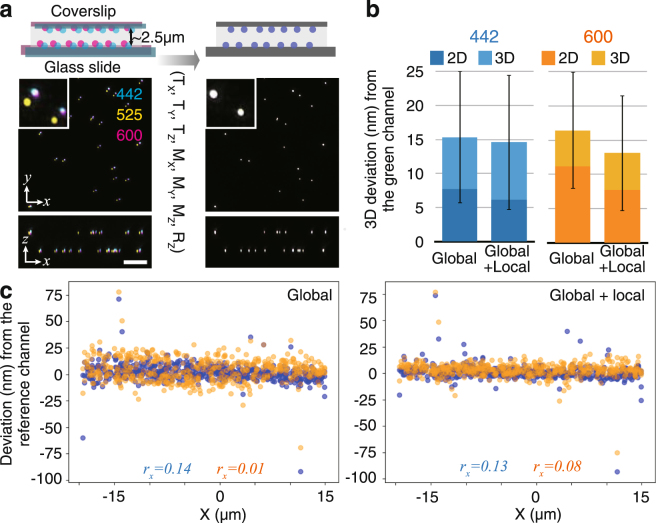
Registration precision. (**a**) A 3D-SIM image of multicolor beads immobilized both on the coverslip and on the glass slide before (left) and after (right) registration. Maximum projections of the quarter field of view (20 × 20 × 8 µm) along the XY plane and the XZ plane are shown with a region magnified in the inset. The blue, green, and orange channels are indicated by their respective central emission wavelengths. Scale bars represent 5 µm for the larger field of view including the vertical (Z) axis and 1.25 µm for the inset. (**b**) Mean distances of individual bead localizations in the blue and orange channels relative to the green reference channel, applying the ‘global’ and ‘global + local’ registration methods. The 3D positions of 527 beads on two-layer multicolor bead samples were determined by 3D Gaussian fitting, and the 3D distances were averaged. Error bars indicate SD. (**c**) Plots of beads along the X coordinate. The Pearson correlation coefficient (*r*) is shown for each channel to show the correlation, if any, between deviation and position in the field of view.

The registration error of the global and local correction methods was measured for real datasets of super-resolution 3D-structured illumination microscopy (3D-SIM) images^20,21^ acquired from two-layer beads samples. These two-layer beads samples were prepared by immobilizing multispectral fluorescent beads on the surfaces of both the coverslip and the glass slide, separated by ~2.5 µm of mounting medium^22^ (Fig. 4a). Thus, we acquired image stacks of these two-layer beads through 3D-SIM in three channels. Then, their relative chromatic shifts were measured and corrected by the global and local registration methods, as explained above. Thereafter, the centroid coordinates of individual beads were determined through 3D elliptical Gaussian fitting for each channel. The deviation of the coordinates in the blue and orange channels from the reference (green) channel of each bead were measured. We assumed that the distance of coordinates between channels become zero if the registration was perfect, and any deviation from zero was regarded as registration error. The resulting registration error for the two bead’s layers was 15.3–16.4 nm in 3D XYZ space and 7.7–11.2 nm in the 2D XY plane when only the global registration method was used (Fig. 4b). There was negligible correlation between registration precision and the horizontal distance from the center of the field of view (Pearson correlation coefficient *r* = 0.01 to 0.14 along the X-axis, Fig. 4c, and −0.26 to 0.15 along all three axes, Supplementary Fig. S5).

We also examined the effects of local correction and global correction. The 3D registration accuracy actually improved to 12.4–14.0 nm in 3D XYZ space (Fig. 4b,c, Supplementary Fig. S5) and 5.3–6.9 nm in the 2D XY plane. Thus, registration accuracy improved with local corrections and now satisfies the increased requirements of high relative localization accuracy that come with most super-resolution applications.

Our approach using any kind of image as a reference for channel alignment enabled a simple calculation workflow, making the method uniformly accomplishable by a single piece of software. This workflow was implemented in a stand-alone cross-platform software application named *Chromagnon*, which provides an intuitive graphical user interface and is suitable for use with various microscopy file formats. The source code was written in Python and the software is available at: https://github.com/macronucleus/chromagnon. Our software enables the user to determine the alignment parameters in reference images and apply them to multi-dimensional (x, y, z, λ, t) target images (see Supplementary Fig. S6).

### Accuracy of registration using calibration slides

Variable imaging conditions can influence the chromatic shift in a given sample. To evaluate registration accuracy, using *Chromagnon*, we examined how much uncertainty in chromatic shift is present when using calibration slides. We imaged the calibration slides of two-layered multispectral fluorescent beads (Fig. 4a) using 3D-SIM under various imaging conditions and then quantified the chromatic shifts by measuring the deviation of alignment parameters in each channel. The experimental conditions used in this study are summarized in Supplementary Table S2.

**Figure 5:**
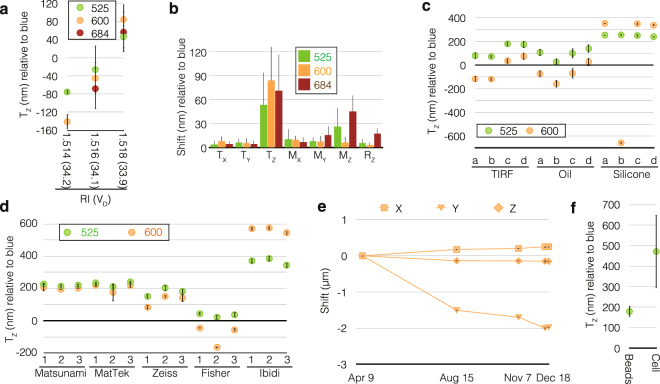
Possible chromatic shifts in calibration slides. (**a**) Translation along the Z-axis (T_Z_) measured for the different refractive indices of immersion oils using the blue channel as a reference. Two-layer multicolor bead slides were imaged by 3D-SIM using immersion oils with different refractive indices as indicated. Bars represent the standard deviation (SD) of 1–3 measurements at different positions of the two slides. The Abbe numbers (v_D_) for these immersion oils are also shown in parenthesis. (**b**) The same data in (**a**) shown for each individual parameter. Larger values indicate greater fluctuation. (**c**) A single set of four two-layer blue bead slides (**a**–**d**) imaged with different objective lenses in the blue, green, and orange channels by deconvolution microscopy; the blue channel was used as a reference to measure T_Z_. Bars represent SD from measurements taken at 5–6 positions of each coverslip. (**d**) Translation along the Z axis measured for three coverslips (numbered as 1, 2, and 3) from different companies. All coverslips were of standard thickness No. 1.5 (0.16–0.19 mm) except for Zeiss No. 1.5 H (0.170 ± 0.005 mm) and Fisher No. 1 (0.13–0.17 mm). Multicolor beads were imaged by 3D-SIM using a silicone immersion objective lens, except for coverslips from Ibidi, for which the results from deconvolution are shown since beam polarization (required for 3D-SIM) was affected by these plastic coverslips. Error bars represent SD from 3–7 acquisitions at different positions on each slide. (**e**) Translation of the orange channel position relative to the blue channel (detected on separate cameras) monitored over eight months. Error bars (horizontal lines) indicate the negligible SD of sample measurements within the same day. (**f**) T_Z_ measured for beads and biological samples using silicone immersion objective lens observed in the blue (442 nm) and green (525 nm) channels. “Cells” indicates fixed biological samples on the coverslip imaged by 3D-SIM at a mean observation depth of ~3 µm. Bars represent SD from at least three different slides.

First, we examined the chromatic shift due to differences in spherical aberration by changing the refractive index of the immersion oil by using the same slide. When the refractive index of the immersion oil was changed by as little as 0.002, translation along the Z axis (T_Z_), which represents the axial chromatic aberration, varied by as much as ~130 nm (Fig. 5a). As shown in Fig. 5b, T_Z_ was generally the parameter most affected by spherical aberration when high numerical aperture (NA) objective lenses were used (e.g., NA 1.40 in our experiments). In contrast, transverse chromatic aberration (represented by M_X_ and M_Y_ in Fig. 5b) was only minimally influenced by imaging conditions at the size of the imaging field used in this experiment (512 × 512 pixels measuring ~40 µm in real space).

Secondly, using a set of four slides (named ‘a–d’ in Fig. 5c) of two-layer multispectral bead samples, we compared different objective lenses. Interestingly, even for each single sample (e.g., ‘b’), T_Z_ was highly variable, by as much as 720 nm when using different objective lenses (Fig. 5c). Furthermore, the chromatic shift varied by as much as ~1 µm between different samples using the same objective lens (Fig. 5c). Variation of T_Z_ in each coverslip, represented by the error bars, was generally negligible (Fig. 5c).

Thirdly, we compared different types of coverslips from various companies. The registration parameters were again measured with fluorescent beads (Fig. 5d). T_Z_ varied by as much as 740 nm between different coverslip types (Fig. 5d). The differences in T_Z_ were negligible among coverslips from some companies, but significant between coverslips from different companies (Fig. 5d). Variation of T_Z_ within each coverslip was again very small (shown as the error bars in Fig. 5d).

Furthermore, because our microscope was equipped with multiple cameras, we measured shifts in the registration parameters for these cameras; the measurements were done over a period of eight months. The registration parameters drifted by as much as 2 µm in eight months (Fig. 5e).

Finally, we compared T_Z_ of single-layered bead samples and fixed cultured mammalian cells under the same conditions. Images were obtained through 3D-SIM. The values of T_Z_ measured with the cells differed greatly from those of the bead samples by as much as ~440 nm (Fig. 5f). This justifies the reason why chromatic shift has to be measured in cells.

Based on these data, the uncertainty of the chromatic shift when using calibration slides is summarized in Table 1. Bead-based calibration slides may introduce all kinds of uncertainty if the possible causes of chromatic shifts are not carefully taken care of. Biological calibration slides reduced the shift due to differences in the dispersion of samples, but other uncertainties still remained the same as in bead-based calibration slides (as presented in Table 1).

**Table 1:** Method-dependent uncertainty ranges of chromatic shifts.

Origin of Shift	Methods				
	Calibration slide (beads)	Calibration slide (biological)	Cross-talk on demand	Replicates on the coverslip	Itself
3D alignment by *Chromagnon*	13–17 (14)	13–17 (14)	13–17 (14)	13–17 (14)	13–17 (14)
Dispersion of samples	6–440 (100)	6–30 (15)	6–700 (40)	6–30 (15)	0
Difference in coverslips	0–1000 (40)	0–1000 (40)	0	0	0
Difference in spherical aberration	0–225 (100)	0–225 (100)	0	0	0
Mechanical drifts	0–2000 (100)	0–2000 (100)	~0	~0	0
Total	20–3682 (354)	20–3272 (269)	19–717 (54)	19–47 (29)	13–17 (14)
**Recommended microscopy technique**	TIRFM, SMLM	CLSM, STED, SIM	WFM, SMLM	CLSM, STED, SIM	

Values are typical minimum-maximum (mean) values, expressed in nm, determined from all the data presented in this study. The origins of the shifts depend on the imaging methods and may not be applicable to certain microscopy modalities. Abbreviations: TIRFM, total internal reflection fluorescence microscopy; SMLM, single-molecule localization microscopy; CLSM, confocal laser scanning microscopy; STED, stimulated emission depletion; SIM, structured illumination microscopy; WFM, wide-field microscopy.

### Accuracy of registration using cross-talk on demand

**Figure 6:**
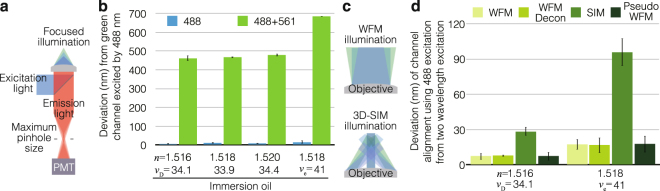
Chromatic shift in illumination. (**a**) An illustration of the CLSM setup to measure chromatic shift only for illumination. When the pinhole was opened to its maximum size, almost all light goes into the detector (PMT). Therefore, the chromatic shift of emission light is negligible and only that of the illumination light is measured. (**b**) Fixed cells were stained with phalloidin conjugated with Alexa 488 and 594. The green channel, obtained by exciting with 488 nm, was aligned with respect to the red channel, obtained by exciting with 488 (“488” blue bars) or 561 (“488 + 561” green bars) nm using the global method. The vector sum of the seven global alignment parameters is shown. Different immersion oils were used to examine the influence of spherical aberration and dispersion. (**c**) An illustration of WFM and 3D-SIM illumination around the sample. (**d**) The same samples from (**b**) were imaged with WFM or 3D-SIM using the same objective lens. WFM images were deconvolved (WFM Decon) and 3D-SIM raw images were averaged to create pseudo-WFM images. The red channel excited with 488 nm and the green channel excited with 488 nm were aligned with respect to the red channel excited with 561 nm and the green channel excited with 488 nm. The vector sum of the seven alignment parameters is shown.

Next, we measured chromatic shift when using the cross-talk on demand approach. In this approach, differences in the excitation wavelengths for the reference and target images (see Fig. 1c,e) may introduce chromatic shift, even though they are detected at the same emission wavelength. Thus, we measured the chromatic shift of the excitation wavelength by using confocal laser scanning microscopy (CLSM). With the pinhole opened to its maximum setting, almost all the incoming light is detected and only the excitation wavelength affects chromatic shift (Fig. 6a). We prepared fixed cell samples stained with green and red phalloidin and measured the seven global alignment parameters due to chromatic shift between the green and red channels using a set of immersion oils with varying optical properties. When using only a single excitation wavelength (488 nm) for the green and red channels, the chromatic shift was within the range of the measurement precision (4.8–14.6 nm). However, when using the respective excitation wavelengths (488 nm and 561 nm) for the green and red channels, the chromatic shift was very large (470.8–687.3 nm), as shown in Fig. 6b. Using immersion oils with different optical properties also revealed that chromatic shift depends more on dispersion rather than the refractive index of the immersion oil, which is consistent with the assumption that the chromatic aberration of the excitation wavelength was the main cause for the observed shifts.

Microscopy methods using focused illumination, including 3D-SIM, may well be influenced by different excitation wavelengths. On the other hand, illumination in wide-field microscopy (WFM) is hardly influenced by different excitation wavelengths (Fig. 6c). Thus, we used the same samples and objective lenses as in the CLSM experiments described above and imaged with WFM and 3D-SIM. Then, the seven global alignment parameters due to chromatic shifts of images taken using either a single excitation wavelength (488 nm) or two excitation wavelengths (488 nm and 561 nm) were compared using the global method. The chromatic shifts obtained through WFM with and without subsequent deconvolution were within the range of the measurement precision (7.4–17.5 nm), while those obtained through 3D-SIM were much larger (28.4–95.9 nm), as shown in Fig. 6d. Experiments using two different immersion oils showed that the chromatic shifts were again more pronounced for certain dispersion properties. Interestingly, creating pseudo-WFM images from raw 3D-SIM images by computationally averaging the structured illumination removed all the chromatic shifts in 3D-SIM (Fig. 6d). These data indicate that 3D-SIM was influenced by the chromatic aberration of the excitation light, while WFM was not.

Since bright-field images can be obtained in all channels, we also examined if bright-field images can be used for channel alignment similarly to the cross-talk on demand approach (Supplementary Fig. S7a). The global registration parameters from bright-field images, however, deviated from the references obtained with WFM (58.4–65.3 nm, Supplementary Fig. S7b), and the deviation was larger than the computational uncertainty (approximately 16 nm). Therefore, bright field images may be used as registration references albeit with a lower accuracy.

Based on these data, we concluded that the uncertainty of the chromatic shift when using the cross-talk on demand method depends on the microscopy method used, and this is summarized in Table 1.

### Accuracy of registration using replicates on the same coverslip

**Figure 7:**
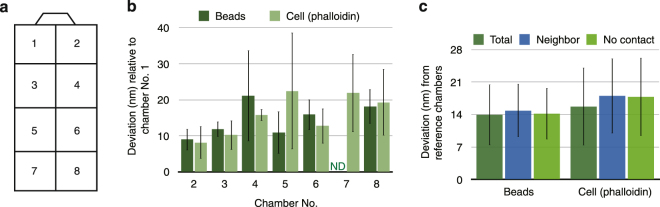
Chromatic shifts of replicates on the same coverslip. (**a**) Illustration of a chambered coverglass with numbers temporarily assigned to individual chambers. (**b**) Multispectral fluorescent beads and fixed cells stained with phalloidin conjugated with Alexa 488 and 594 were imaged with 3D-SIM. Then, the vector sum of the global alignment parameters for green and red was compared with chamber No. 1. (**c**) Mean difference of the vector sum of the global alignment parameters with chamber No. 1 (“Total”), neighboring chambers (“Neighbor”), and chambers with no contact (“No contact”).

As documented above, both the calibration slides and the cross-talk on demand approaches had weaknesses, particularly for advanced imaging modalities. Therefore, we devised a new method to measure the chromatic shift for biological samples. From the measurements we presented in Fig. 5c,d, the chromatic shifts were very similar within a single coverslip. Thus, it would be ideal if both the sample of interest and the biological calibration samples were placed on a single coverslip. We propose a new method with replicates on the same coverslip, utilizing commercially-available chambered coverglasses (Fig. 7a). Chambered coverglasses consist of a single coverslip divided into several partitions by plastic walls. We prepared single-layered multispectral beads and fixed cell samples stained with green and red phalloidin, and imaged them by 3D-SIM. For both samples, the mean chromatic shift measured using the global method was only 14.0–15.8 nm from one partition to the other within each single chambered coverglass (Fig. 7b). We examined if the nearest neighbor (the one with maximum contact, e.g., Partition 3 and Partitions 1, 4, and 5 in Fig. 7a) presented any advantage for obtaining global registration parameters over distantly located partitions (those with no direct contact, e.g., Partition 3 and Partitions 7 and 8 in Fig. 7a). No statistically significant difference was found between distantly located partitions and neighboring partitions within the precision limitations of the global registration method (Fig. 7c, Student’s *t*-test, *p* = 0.71–0.94).

The uncertainty in chromatic shift when using replicates on the coverslip according to our data is summarized in Table 1.

## Discussion

Our calculation approach measures global registration (affine) parameters by extending conventional methods of phase correlation, making it easier to perform chromatic correction using any type of biological sample, not limited to point-like objects. Calculations based on feature detection^16^ require very distinct features (e.g., fluorescent beads), which are not always present in biological images. Log-polar transformation and optimization-based methods could be used for biological images, but, from our experience, these were not sufficiently accurate for use in super-resolution microscopy. The presented quadrisection phase correlation method is more robust, fast, and accurate, and its accuracy is further enhanced by combining it with local registration. The overall registration precision after local registration was 5.3–6.9 nm in 2D space and 12.4–14.0 nm in 3D space. These values were comparable to the registration precisions obtained with the localization of multispectral beads^6,9–11^. Although our method does not set the highest benchmark in the literature (e.g., Gahlmann *et al*. attained a mean 3D fiducial registration error of 7.84 nm^10^), it should be noted that our registration method is able to measure chromatic shift from biological images. Our simulation showed that quadrisection phase correlation worked even at an average SNR of <1.5 in each optical section, providing the possibility of reducing illumination intensity to obtain registration parameters from living cells. Whereas previous registration approaches based on feature detection require optimization for individual experiments, our workflow is simple enough to implement chromatic alignment into an easy-to-use software solution. Our multi-platform open-source *Chromagnon* software is freely available with a user-friendly, stand-alone interface, and can be applied to a wide variety of multi-channel fluorescence microscopy image formats, including 2D, 3D, time-lapse, conventional, and super-resolution image data. Notably, while quadrisection phase correlation works well for aligning multicolor microscopy images (rotation was <3° and the magnification factor was <1.1 in fluorescence microscopy; see Supplementary Table S1), it is limited in the possible amount of rotation and magnification, which should be sufficiently small to measure the phase correlation of the quadrants clearly. Thus, when working with images with larger rotation and magnification, calculations should be preceded by other methods, such as the log-polar transformation, and only the final values should be measured using quadrisection phase correlation.

Our quantitative measurements evaluated how much uncertainty in the chromatic shift was to be expected using different methods due to imaging conditions, samples, and mechanical drifts, and our results are summarized in Table 1. However, it is important to note that some causes of chromatic shift might not take effect depending on the experiment. For example, mechanical drift is not applicable for single-camera setups. Similarly, the level of spherical aberration may be constant if the sample type, the refractive index of the embedding medium, and temperature are kept constant. Hence, our recommendations are shown at the bottom of Table 1 as a guide to choose which registration method should be used for a given microscopy technique. The cross-talk on demand approach is the preferred method for WFM and related microscopy techniques, while for microscopy techniques that use focused or tilted illumination, such as CLSM and 3D-SIM, using replicates on the same coverslip provides more accurate chromatic shift correction. Alternatively, for convenient repeated usage, a dedicated calibration slide can be prepared using carefully controlled imaging conditions reproducible between the calibration and sample slides. A calibration measurement should be recorded at the beginning of each imaging session, and the corresponding registration parameters then applied to all subsequent experiment acquisitions within the session. The various registration methods implemented in the *Chromagnon* software (summarized in Table 1) will simplify and speed up multicolor imaging analyses at higher 3D registration precisions even for non-specialized researchers.

## Methods

### Sample preparation for imaging

Immunostaining of HeLa cells was conducted as previously described^23^ with slight modifications. After fixation and permeabilization, the cells were stained with indirect immunofluorescence. The primary antibodies and their dilution ratio were as follows: anti-treacle monoclonal antibody (2–30C, 1:1000, which was a generous gift from Dr. Masahiro Kumeta, Kyoto University)^24^, anti-fibrillarin monoclonal antibody (1:250, Cytoskeleton Inc.), anti-α-tubulin monoclonal antibody (DM1A, 1:1000, Sigma-Aldrich), and rabbit polyclonal anti-lamin B1 (1:1000, Abcam). A secondary antibody, labeled with either Alexa Fluor dyes (Thermo Fisher Scientific Inc.) or CF405M dye (Biotium), was used at 1:500 dilution. Alexa Fluor 488 Phalloidin and Alexa Fluor 594 Phalloidin (Thermo Fisher Scientific Inc.) were used at 0.4 units/ml. The cells were counterstained with either DAPI or Hoechst 33342 at 100 ng/ml, and mounted with ProLong Diamond Antifade Mountant (Thermo Fisher Scientific Inc.) or VectaShield Mounting Medium (Vector Laboratories). For dual-labeling of treacle and fibrillarin, an anti-fibrillarin monoclonal antibody was conjugated with Alexa Fluor 546 using the Zenon Labeling Kit (Thermo Fisher Scientific Inc.) and served as third antibody. The cells were stained with DAPI at 100 ng/ml, and then mounted with ProLong Gold Antifade Mountant (Thermo Fisher Scientific Inc.). The samples were observed immediately after mounting.

Multispectral fluorescent beads (TetraSpeck Microspheres, 0.2 µm, Thermo Fisher Scientific Inc.) were resuspended in absolute ethanol at a dilution ratio of 1:9. Alternatively, blue fluorescent beads (FluoSpheres 1.0 µm F13080, Thermo Fisher Scientific Inc.) were resuspended in ethanol at a dilution ratio of 1:19. Without delay, 5 µl of each bead suspension were spread on clean 18 × 18 mm coverslips of different types (No. 1 S, Matsunami; No. 1.5 H, Zeiss; 35-mm glass bottom dishes MatTek Cat# P35G-1.5-14-C; 18 Well µ-Slide Ibidi Cat# 81826). Alternatively, 7.5 µl of the multispectral fluorescent bead suspension was spread on a 25 × 25 mm coverslip (No. 1, ThermoFisher Scientific Inc. Cat# 12-548-B). We used 100% glycerol (absorptiometric-analysis grade; Wako) with 4% n-propyl gallate (pH 7.0) as mounting medium for the oil immersion objective lens and a 1:1 dilution of this solution with phosphate buffered saline (with an expected refractive index of 1.40)^25^ for the silicone immersion objective lens. The stock solution (100% glycerol with 4% n-propyl gallate) before pH adjustment was stored at −30 °C, and the aliquots were pH adjusted every time before use. The coverslip was dried, and 1.35–3 µl of the mounting medium was added followed by mounting on a glass slide (0.8–1.0-mm thick, Matsunami) where fluorescent beads were immobilized similarly to the coverslips. The spaces between the coverslips and the glass slides measured 2–20 µm.

When making replicate reference samples on the same coverslip, chambered coverglass (Nunc Lab-Tek II, 8 wells) was used.

### Image acquisition and reconstruction

3D-SIM and wide-field imaging were performed using a DeltaVision OMX V3 microscope and an SR microscope (GE Healthcare) equipped with either multiple Cascade II 512 EMCCD cameras (Photometrics) or edge 5.5 sCMOS cameras (PCO) using either an oil immersion objective lens (UPLSAPO 100XO NA1.40 or PLAPON 60XO NA1.42; Olympus), a TIRF objective lens (UAPON 100XOTIRF NA1.49; Olympus), or a silicone immersion objective lens (UPLSAPO 100XS NA1.35; Olympus). The optical setup is shown in Supplementary Fig. S8. For 3D-SIM with the silicone immersion objective lens, we used a relay lens (f = 70 mm, SigmaKoki) after the diffraction grating, with the correction ring of the objective lens carefully adjusted each time using the green channel as a reference. The detection color channels covered blue (419–465 nm), green (500–550 nm), orange (582–619 nm), red (602.5–655.5 nm), and deep red (665–705 nm) emission ranges, with excitation laser lines of 405, 488, 561, 593, and 640 nm, respectively. The temperature around the sample stage was ~27 °C ± 1 °C. The refractive indices (at 23–25 °C) and the Abbe numbers of the immersion oils used in this study were 1.514 (*v*_D_ = 34.2, Cargille), 1.516 (*v*_D_ = 34.1, Cargille), 1.518 (*v*_D_ = 33.9, Cargille), 1.520 (*v*_D_ = 34.4, Cargille), and 1.518 (*v*_e_ = 41, Olympus). Reconstruction of 3D-SIM images was performed using softWoRx (GE Healthcare) with Wiener filter constants between 0.002 and 0.004. For constrained iterative deconvolution, the Priism suite (http://msg.ucsf.edu/IVE/) was used with a Wiener filter enhancement of 0.9 and 15 iterations.

For CLSM, an LSM 880 microscope (Zeiss) was used with an oil immersion objective lens (UPLSAPO 60XO NA1.42; Olympus) connected using the adaptor for Olympus objective lenses (Zeiss) with the above-mentioned immersion oils. The detection color channels covered the green (490–553 nm) and orange (571–677 nm) emission ranges, with excitation laser lines of 488 and 561 nm, respectively. The pinhole was set to its maximum size (599 µm).

### Global image registration for fluorescence microscopy

For each reference multicolor image, we set a reference channel and subject channels; the subjects were transformed to merge onto the fixed reference. The sections with a strong contrast in the 3D images were selected and maximum-intensity projected along either the Z or X axes. These projection images were used to identify the registration parameters for either the lateral or vertical directions, respectively. The resulting 2D images prepared from the reference and subject images were split into four regions, and phase correlations between the reference and the subject images were calculated for each quadrant (Fig. 3a,b). Phase correlation, which is a modification of cross correlation, was performed as follows. Assuming that $\tilde{S}$ is the Fourier transform of image *S* of channel *λ* to be examined, then the phase information of $\tilde{S}$, denoted as $\tilde{P}$, was obtained as

1 $$\tilde{P}(\lambda ,u,v)=\frac{G(u,v)\tilde{S}(\lambda ,u,v)}{|\tilde{S}(\lambda ,u,v)|}$$

where $|\tilde{S}|$ denotes the amplitude of $\tilde{S}$ and *G* represents a Gaussian mask with SD set at 0.2 of the Nyquist sampling criterion for the highest frequency of the sampling rate of the image, to attenuate frequencies higher than the resolution limit. Then, phase correlation images (*R*) of two channels were obtained as

2 $$R(x,y)={ {\mathcal F} }^{-1}[\tilde{P}(1,u,v){\tilde{P}}^{\ast }(2,u,v)]$$

where ${\tilde{P}}^{\ast }$ represents the complex conjugate and ${ {\mathcal F} }^{-1}$ is the inverse Fourier transform. The difference in the coordinates of the peak correlation from the image center of *R* represents the amount of translation for each quadrant (Fig. 3b,c). Assuming that the center of rotation and magnification is the center of the 2D image—and that only affine parameters, consisting of translation, rotation, and magnification, are involved in the transformation—we can obtain affine parameters as a solution to the simple equations shown in Fig. 3c, as explained in the Results section and the legend to Fig. 3. The rotation vector was converted into an angle by taking arctangent of the vector. The magnification vector was converted into a magnification factor calculated as $\frac{{{\bf{M}}}_{{\bf{xy}}}+{c}_{xy}}{{c}_{xy}}$, where *c*_*xy*_ is the 2D coordinate of the image center. The resulting affine parameters were then applied to the subject images with a third-order spline interpolation using the “affine_transform” function of the “Scipy” package (http://www.scipy.org). Because this function is only able to process 2D sections, the vertical (ZY) axis was processed first, and then the horizontal (XY) axis was processed (see Supplementary Fig. S6). Usually, a single calculation was not sufficient for high-precision image registration; therefore, we iterated the above-mentioned operation until the difference between the current parameter and the last parameter obtained from the previous iteration became less than 1 nm in real space (see the subsection “Conversion from global registration parameters to registration deviation in real space” below) or when the number of iterations reached 20. For the vertical direction, some 3D images did not have enough optical sections for splitting into four regions. In this case, only the translation on the Z axis was corrected using conventional phase correlation. After the global alignment process, the 3D cross correlation was measured to correct the overall 3D translation.

### Local image registration

To measure local distortion, we used a modified version of a previously reported method^19^, as shown in Fig. 3d,e. We started by splitting images into 2 × 2 regions and measured quadrisection phase correlation to obtain the affine parameters. Then, we assumed residual local distortion could be corrected by translation of local parts of the image, that is, if *S* is the image already registered with the affine transformation described above and *U* is the image to be corrected for distortion,

3 $$U^{\prime} (x,y)=S^{\prime} (x+{t}_{x},y+{t}_{y})$$

where $S^{\prime}$ and $U^{\prime}$ are local parts of *S* and *U*, respectively. To find the amounts of translation *t*_*x*_ *and t*_*y*_ in different image areas, the images were split into 4 × 4 elements, and the local phase correlation with the reference channel was measured for each element.

Fluorescence microscopy images often contain regions where contrast is too low to find the peak of phase correlation (Fig. 4e). Therefore, we used the element only if the amount of variance was above a certain threshold. The threshold *v* was determined for an image *S*(*λ*) using the following formula:

4 $$v=c\cdot var(S(1))\cdot var(S(2))$$

where *var* is variance and *c* is a constant for which we used an empirically determined value of 0.1. Then, the variances of the elements *v*_*i*_ were estimated using the following formula:

5 $${v}_{i}=\frac{var(S^{\prime\prime} (1,x,y))+var(S^{\prime\prime} (2,x,y))}{2}$$

where $S^{\prime\prime}$ is the central quarter region of element $S^{\prime}$. The reason why we limited the image regions to the central quarter region (Supplementary Fig. S9a) is because only the center of the element contains the information of pure translation of the region, while peripheral regions of the element may be mixed with the shift values of adjacent regions. This region mask, however, leads to peripheral regions being excluded from shift measurements, even though significant signal amounts may be present. To include these peripheral subregions, we repeated the process with the starting coordinates shifted to half the number of pixels of the elements in either the X or Y axis, or in both the X and Y axes (Supplementary Fig. S9c). Thus, images were split into 4 × 4 regions, but the number of regions examined was 8 × 8 (Supplementary Fig. S9e shows an example of 7 × 7 regions become 14 × 14 regions). Thereafter, the phase correlation of the elements was measured for these regions (Supplementary Fig. S9f). To control the quality of the measured phase correlation, we used results only from elements with a peak value of phase correlation higher than that of an empirically determined value of 0.02. Because these quality controls create empty regions without translation values, they were filled with a special mean filter that only alters the values of empty regions, with a window size that was iteratively increased starting from 3 × 3 regions.

The local registration map obtained in this way was magnified to the respective image size (e.g., 512 × 512) by first applying the Fourier transform, then padding to the image size, and finally applying the inverse Fourier transform to obtain the local registration map *L*(*x*, *y*) of the appropriate image size. To merge affine transformation parameters, a coordinate map *I*(*x*, *y*) corresponding to the image size was affine transformed to obtain the affine registration map *A*(*x*, *y*) using the following equation:

6 $$A(x,y)=I[\frac{\cos \,\theta (x-{x}_{0})+\,\sin \,\theta (y-{y}_{0})}{{m}_{x}}+{t}_{x}+{x}_{0},\frac{-\,\sin \,\theta (x-{x}_{0})+\,\cos \,\theta (y-{y}_{0})}{{m}_{y}}+{t}_{y}+{y}_{0}]$$

where *θ* is the rotation angle around the optical axis, *m*_*x*_ *and m*_*y*_ are the magnification factors along each axis, *t*_*x*_ *and t*_*y*_ are the translation along each axis, and (*x*_0_, *y*_0_) is the rotation center. Then, the total registration map *M*(*x*, *y*) was obtained by adding both maps together: $M(x,y)=L(x,y)+A(x,y)$. The original subject image was transformed by *M*(*x*, *y*) using the “Remap” function of OpenCV (http://opencv.org). Using the resulting image, we iteratively measured local phase correlation and applied it to the subject image as described above until the mean local shift among all regions became <1 nm in real space or when the number of iterations reached 5. Thereafter, the image window size was again halved, and we repeated the above-mentioned operations until the window size reached the minimum size allowed (we used 60 pixels, unless otherwise specified).

### Conversion from global registration parameters to registration deviation in real space

To convert the rotation angle or magnification into the scale used in real space (Figs 2, 6, 7, Supplementary Figs S1, S2, S7), we factored in the lateral pixel doubling of reconstructed SIM images to meet the Nyquist sampling criterion (e.g., 1024 × 1024 × 65 pixels for SIM images and 512 × 512 × 65 pixels for non-SIM images). We used the shift at the edge pixels of such images for calculation purposes. For example, assuming we have 0.5° rotation in a SIM image, the coordinate (1024.0, 512.0) was rotated around the rotation center at (512.0, 512.0). The resulting coordinate (1023.98, 516.47) was subtracted from the original coordinate to obtain the deviation in pixels, (0.02, −4.47). The registration differences due to magnification were calculated similarly. The obtained values were multiplied by the pixel size to obtain deviation in real space. The total registration difference was the vector sum of all seven parameters.

### Estimation of registration accuracy using fluorescent beads

To evaluate the registration accuracy (Fig. 4), two-layer multicolor fluorescent beads each placed on two different positions on two different slides were imaged by 3D-SIM, and the chromatic shift of multicolor images was corrected using the bead image itself. The peak coordinates of individual beads were obtained by 3D Gaussian fitting. Beads at a distance from one another closer than twice the diffraction limit of light were removed from the analysis. The deviation of coordinates from the green channel was determined by subtraction of the coordinates. Total deviation was calculated as the vector sum of the coordinates.

### Simulated tests to measure registration precision

To compare the registration precision of the different methods (Fig. 2 and Supplementary Fig. S1), the deconvolved image stacks (47 sections with 0.25-µm spacing) of either tubulin stained with CF405M or actin stained with Alexa488 were two-dimensionally shifted a known amount (T_X_ = −2.0, T_Y_ = −3.0, T_Z_ = 0, M_X_ = 0.998, M_Y_ = 0.999, M_Z_ = 1.0, and R_Z_ = 0.5, where translations are expressed in pixels, rotation in degrees, and magnifications in zoom factors), and the margins outside the boundaries of the input images were removed. Both channels of the original images were divided by constants ranging from 50 to 500, and noise with a standard deviation of 10 were added to both channels. The noise images were computationally created for each simulation experiment. Maximum intensity projection was used for 2D registration parameter acquisition. SNR was calculated as µ/σ, where µ is the mean of the signal above a visually determined threshold (see Supplementary Fig. S1a) and σ is the standard deviation of noise, which was always 10.

For the log-polar method, translation was first estimated by calculating the phase correlation of the two images as described above. Then, both images were log-polar transformed with rotation angles ranging from 0 to *π* and a log base of 1.012, and the phase correlations between the two images were obtained. The rotation angle and magnification factor were obtained as the coordinates of peak intensity. These operations were iterated until the difference with the previous iteration became less than 1 nm in real space or when the number of iterations reached 20.

For the simplex method, a starting guess was obtained for translation, rotation and magnification. The initial guess for translation was obtained by calculating the phase correlation of the two images as described above. Then, the initial guess for rotation was estimated by rotating one image from −0.4° to 0.4° with a step of 0.02°, removing the margins outside the boundaries of input images after rotation, and then obtaining the Pearson correlation coefficient between the two images for each rotation angle. Afterwards, the values of the Pearson correlation coefficient were fitted with a 6th order polynomial function to estimate the rotation angle that yielded the maximum Pearson correlation coefficient between the two images. An initial guess for magnification along the X and Y axes was also individually estimated by zooming in/out on one image using magnification factors from −0.98 to 1.02 with a step of 0.005, removing margins, and estimating the magnification angles that yielded the highest Pearson correlation coefficient between the two images, as described for rotation. Then, using these estimates as an initial guess, registration parameters were obtained with the simplex algorithm using the ‘fmin’ function of the Scipy package (http://www.scipy.org) by minimizing the returned value of the cost function, which performs (i) affine transformation according to the given registration parameters starting from the initial guess, (ii) removal of the margins of image boundaries, and (iii) returns the inverse value of the Pearson correlation coefficient between the two images.

To examine the average error of the local alignment method (Supplementary Fig. S4), local shifts of a known amount, shown in Supplementary Fig. S4a, were introduced into the actin and tubulin images shown in Supplementary Fig. S1. Then, two channels of the images were divided by constants ranging from 20 to 200, and noise with a standard deviation of 10 was added to both channels, similarly to Supplementary Fig. S1. The maximum intensity projection along the Z axis was used to acquire the 2D local translation map. Regions that contributed to the calculation of local alignment were determined according to equations (4) and (5). A map of these regions was magnified to the respective image size (512 × 512) by first applying the Fourier transform to the map, then padding to the image size, and finally applying the inverse Fourier transform to obtain a region mask of the appropriate image size (Supplementary Fig. S4b). The mean error $(\bar{\partial })$ of the local alignment (Supplementary Fig. S4c) was estimated as

7 $$\bar{\partial }=\frac{{\sum }_{x=1}^{nx}{\sum }_{y=1}^{ny}|L(x,y)-S(x,y)|}{nx\cdot ny}$$

where *L*(*x*, *y*) is the calculated translation map, *S*(*x*, *y*) is the induced shift, *x* and *y* are the coordinates filtered by the region mask, and *nx* and *ny* are the number of pixels along the X and Y axes of the regions filtered by the region mask, respectively.

## Electronic supplementary material

Supplementary Information
