# Supplementary material for: Accurate and fiducial-marker-free correction for three-dimensional chromatic shift in biological fluorescence microscopy
Source: Sci Rep. 2018 May 15;8:7583. doi: 10.1038/s41598-018-25922-7 (PMC5954143; doi:10.1038/s41598-018-25922-7)
Supplement: Supplementary file 1 — Supplementary Information [file 41598_2018_25922_MOESM1_ESM.docx]

**SUPPLIMENTARY INFORMATION**

**Accurate and fiducial-marker-free correction for three-dimensional chromatic shift in biological fluorescence microscopy**

Atsushi Matsuda^1,2 *^, Lothar Schermelleh^3^, Yasuhiro Hirano^2^, Tokuko Haraguchi^1,2^, and Yasushi Hiraoka^1,2 *^

^1^ Advanced ICT Research Institute Kobe, National Institute of Information and Communications Technology, 588-2 Iwaoka, Iwaoka-cho, Nishi-ku, Kobe 651-2492, Japan

^2^ Graduate School of Frontier Biosciences, Osaka University, 1-3 Yamadaoka, Suita 565-0871, Japan

^3^ Micron Advanced Bioimaging Unit, Department of Biochemistry, University of Oxford, South Parks Road, Oxford OX1 3QU, United Kingdom

*Correspondence and requests for materials should be addressed to A.M. (email: a.matsuda@nict.go.jp) or Y. Hiraoka (email: hiraoka@fbs.osaka-u.ac.jp)

**Fig. S1. Comparison of the performance of registration parameter acquisition methods.**


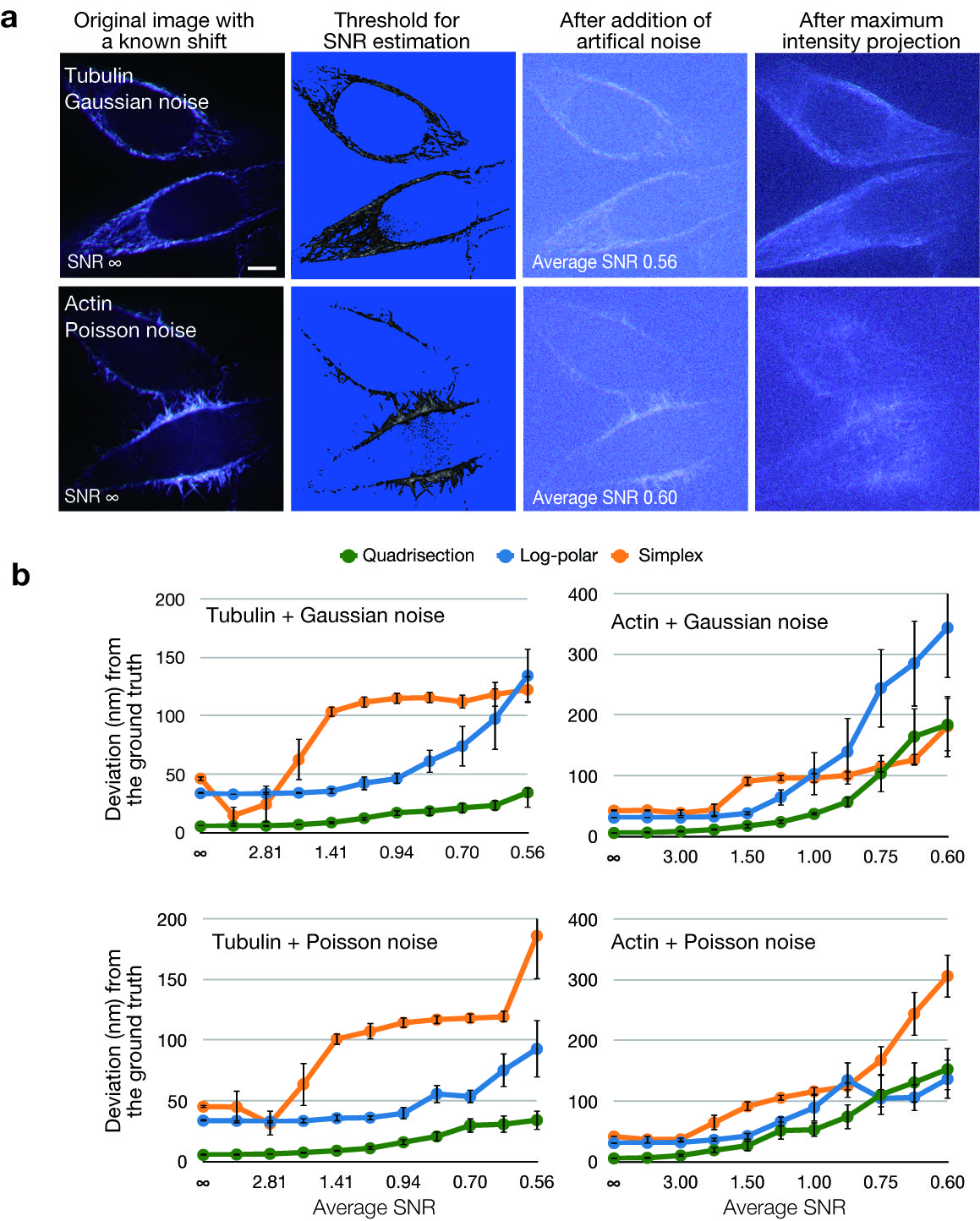


(**a**) The left panels show image stacks of tubulin stained with CF405M or actin stained with Alexa488 were two-dimensionally shifted a known amount (T_X_=−2.0, T_Y_=−3.0, T_Z_=0, M_X_=0.998, M_Y_=0.999, M_Z_=1.0, and R_Z_ =0.5, with translation expressed in pixels, rotation in degrees, and magnification in zoom factor). A magnification difference of 0.001 between the X and Y axes was commonly found in microscopy data (see Supplementary Table S1). Both channels of the original images were divided by constants ranging from 50–500, and computationally created noise images with a standard deviation of 10 were added. A mean value of 0 was used for the Gaussian noise. Maximum intensity projections were created to obtain 2D registration parameters (right panels). Scale bar indicates 5 µm. (**b**) Deviations from the known registration parameters are plotted as a function of the average SNR. The average SNR was calculated as µ/σ, where µ is the mean of the signal above a visually determined threshold (see **a**) and σ is the standard deviation of the added noise (which was 10). Deviation from the known registration parameters, i.e., the vector sum of the five parameters T_X_, T_Y_, M_X_, M_Y_, and R_Z_, is shown. The bars indicate the standard errors of six measurements.

**Fig. S2. Simulation of the effect of unrelated image content on registration errors.**


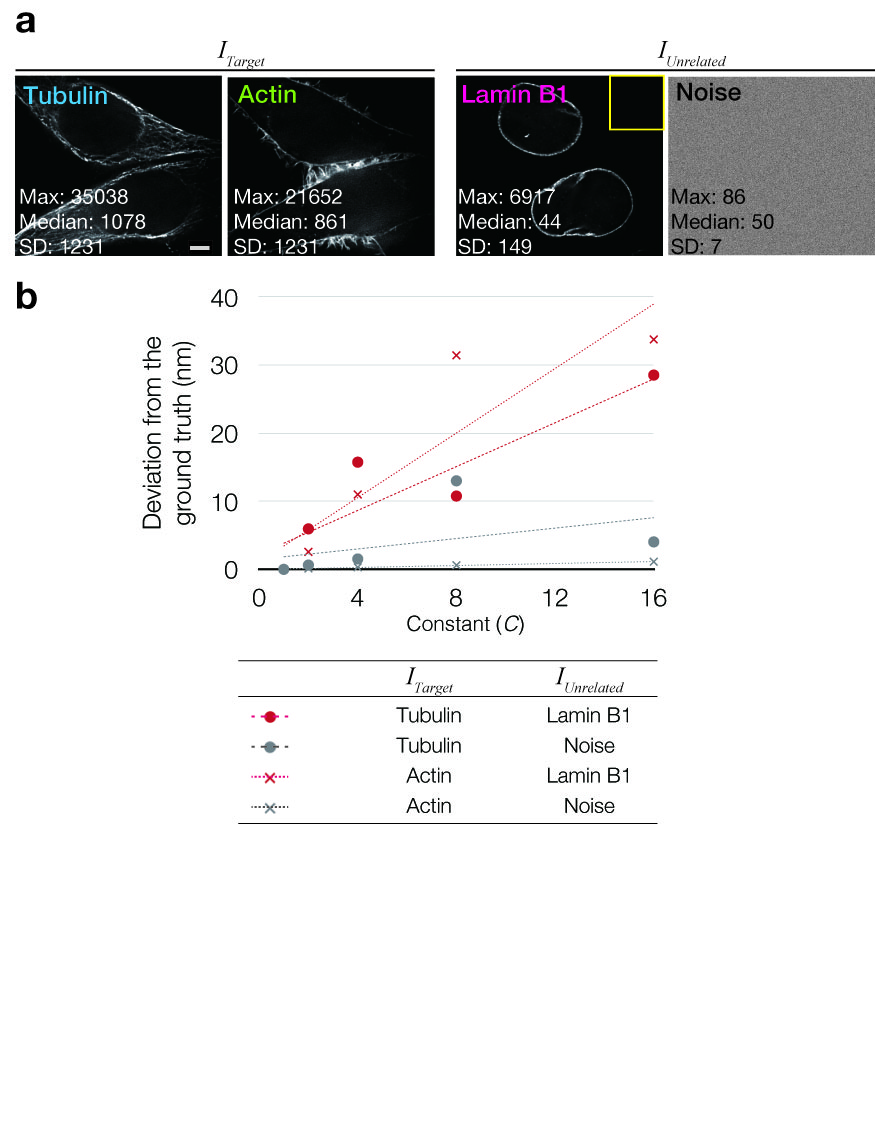


(a) Images of either tubulin or actin ($I_{Target}$) and unrelated images of lamin B1 or noise ($I_{Unrelated}$). The original images of either tubulin or actin were duplicated and one of them was shifted, rotated and magnified a known amount (T_X_=−2.0, T_Y_=−3.0, T_Z_=0, M_X_=0.998, M_Y_=0.999, M_Z_=1.0, and R_Z_=0.5, with translation expressed in pixels, rotation in degrees, and magnification in zoom factor). Then, unrelated images $I_{Unrelated}$ were added to the shifted images $I_{Target}$. Shifted/contaminated images $I_{Modified}$ were generated by the equation $I_{Modified}=\frac{I_{Target}}{C}+\frac{I_{Unrelated}}{2}$, where *C* is a constant that controls the ratio of signal intensity in the image.

(b) Deviation from the ground truth plotted against the constant *C*. The registration parameters between the original ($I_{Target}$) and shifted/contaminated images ($I_{Modified}$) were calculated using quadrisection phase correlation (see Fig. 3), and deviation from the ground truth (i.e., the vector sum of the five parameters T_X_, T_Y_, M_X_, M_Y_, and R_Z_) was plotted against the constant *C*. The Gaussian noise image was computationally created from the noise statistics (mean and SD) in the region bounded by the yellow box in the lamin B1 image. The scale bar is 5 µm.

**Fig. S3. Parameter output from local alignment.**

**
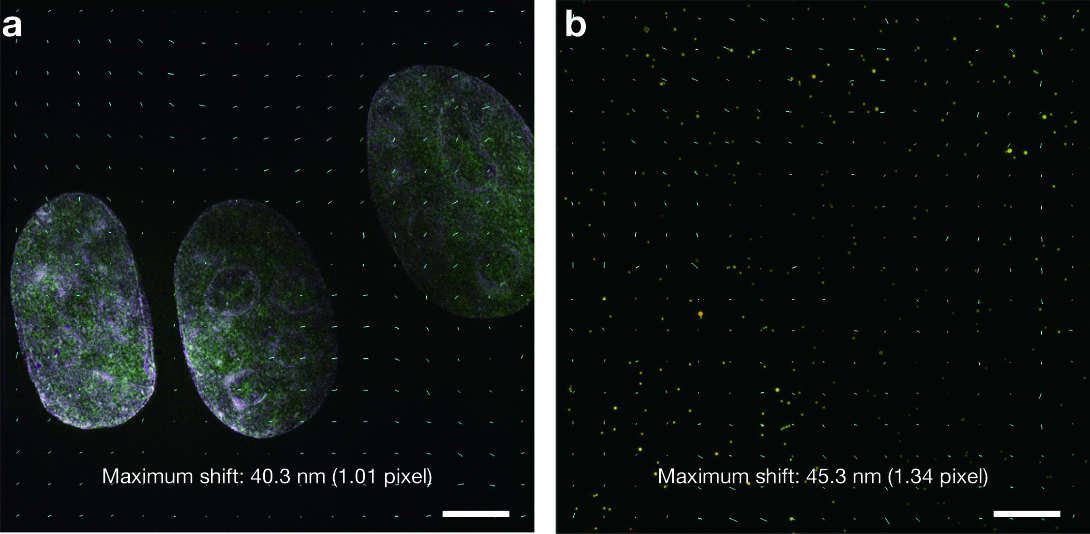
**

(**a**) Local translation vectors for the image shown in Fig. 3d with the minimum window size of 60 pixels. Translation of local pixels is indicated by cyan lines, whose lengths were magnified by a factor of 20 for better visibility. Note that translation vectors in areas without any objects used the mean of the surrounding regions with significant contrasts (see Methods). (**b**) Multicolor beads image with representative translation vectors (20× magnified) in different areas of the field of view. Scale bars are 5 µm.

**Fig. S4. Precision of local registration.**


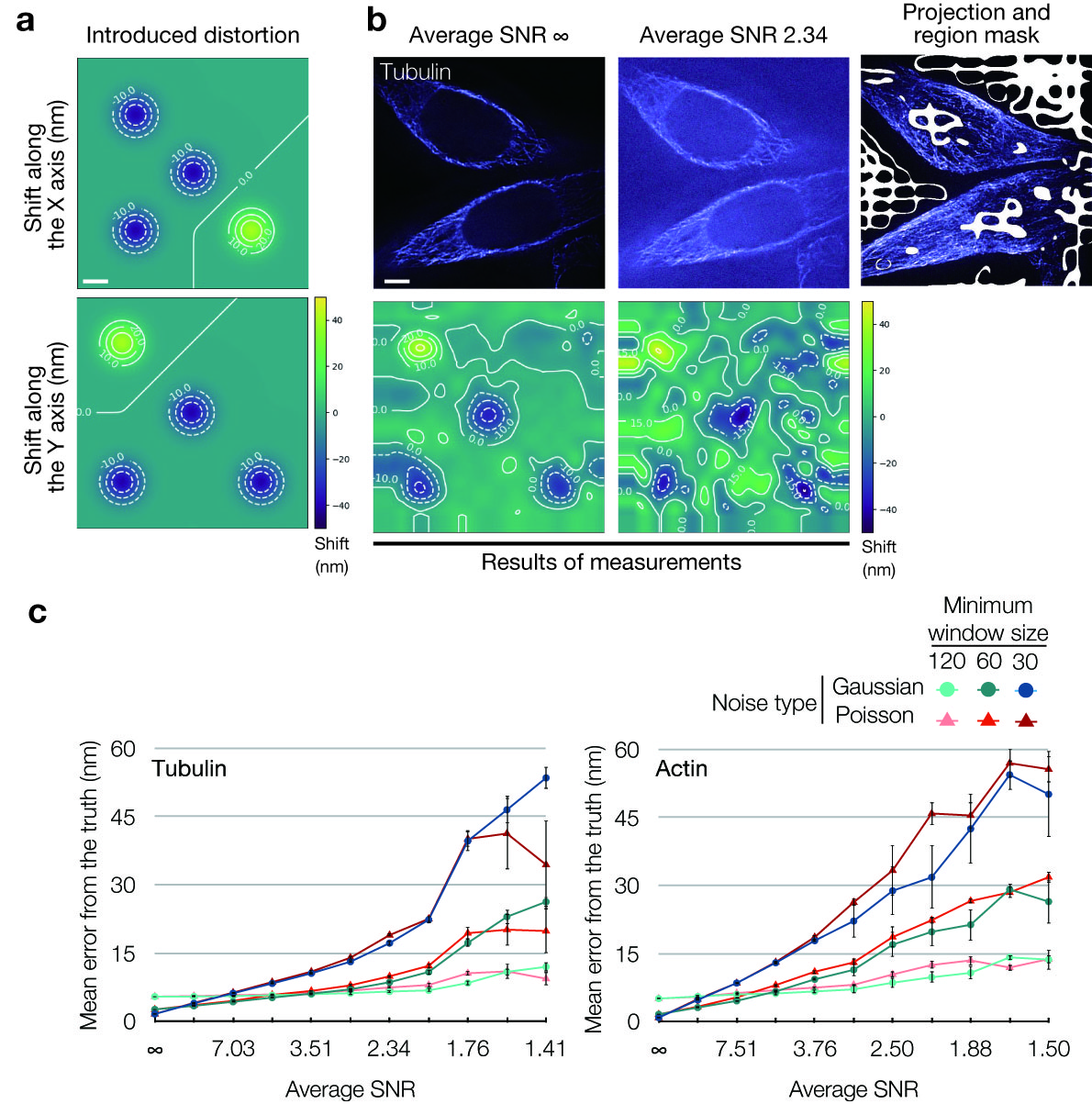


(**a**) Amounts of artificial local shifts along the X and Y axes. The scale bar indicates 5 µm. (**b**) Local shifts were added to the series of microscopic images with simulated noise, similarly to Supplementary Fig. S1. The two panels in the upper row show representative optical sections of a 3D stack image (504 × 504 × 64 pixels) with and without Gaussian noise. The right-most panel shows the maximum intensity projection of the image with an average SNR of 2.34 superimposed with a region mask (white) that excludes regions with low signal variance, while using a minimum window size of 60 × 60 pixels. The mask was distorted by the interpolation process, but helped to exclude most of the pixels that did not contribute to the calculation process. The two lower panels show representative local alignment maps along the Y axis calculated from the maximum intensity projection of the 3D stack shown in the upper panels using a minimum window size of 60 × 60 pixels. See Supplementary Fig. S1 and Method for the creation of simulated images and the estimation of the average SNR. The scale bar indicates 5 µm. (**c**) Plots of the mean deviations from the known shift as a function of the average SNR for two kinds of datasets (“Tubulin” and “Actin”). The window size was iteratively halved, and the processes of calculating local phase correlation and application of the resulting alignment map were repeated until the window size reached the minimum window size in pixels. The regions filtered by the region mask for each window size were used for the calculations (see Methods). The bars indicate the standard errors of six measurements.

**Fig. S5. Deviation of registration in each axis.**


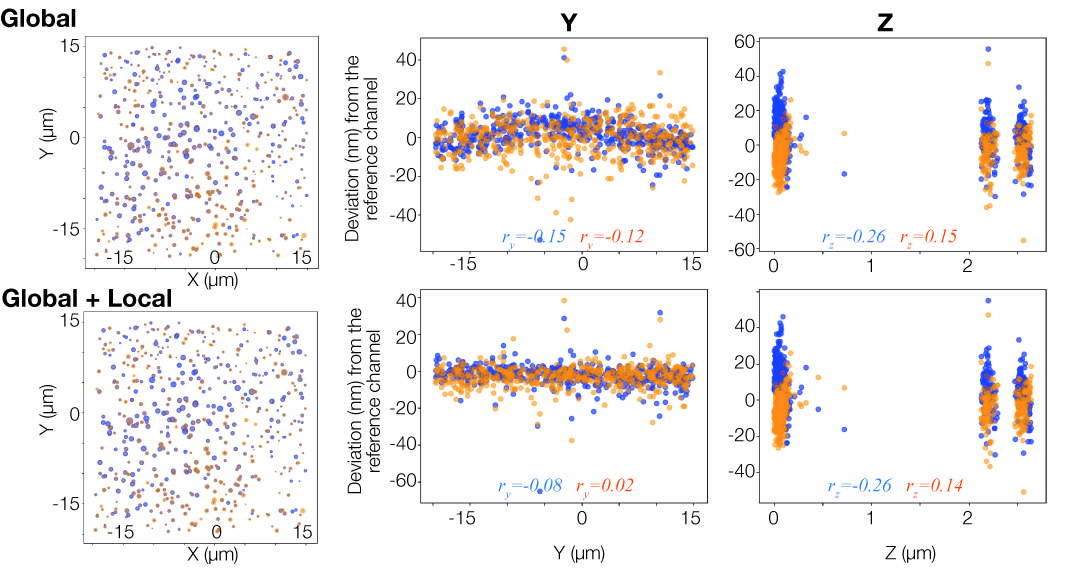


Multispectral beads on two-layer bead slides were imaged in the blue, green, and orange channels. The differences in coordinates of a total of 527 beads after global and local registration are plotted along each axis with the green channel used as reference. For the X and Y axes, the positions of individual beads were plotted with relative dot sizes indicating the amount of translation on the X and Y axes. For the Y and Z axes, the deviation of registration is shown along the respective coordinates with color indicating the blue and orange channels. The Pearson correlation coefficient (*r*) is shown for each channel and each axis to show correlation, if any, between deviation and position in the field of view.

**Fig S6. Schematic of the workflow of our registration software.**


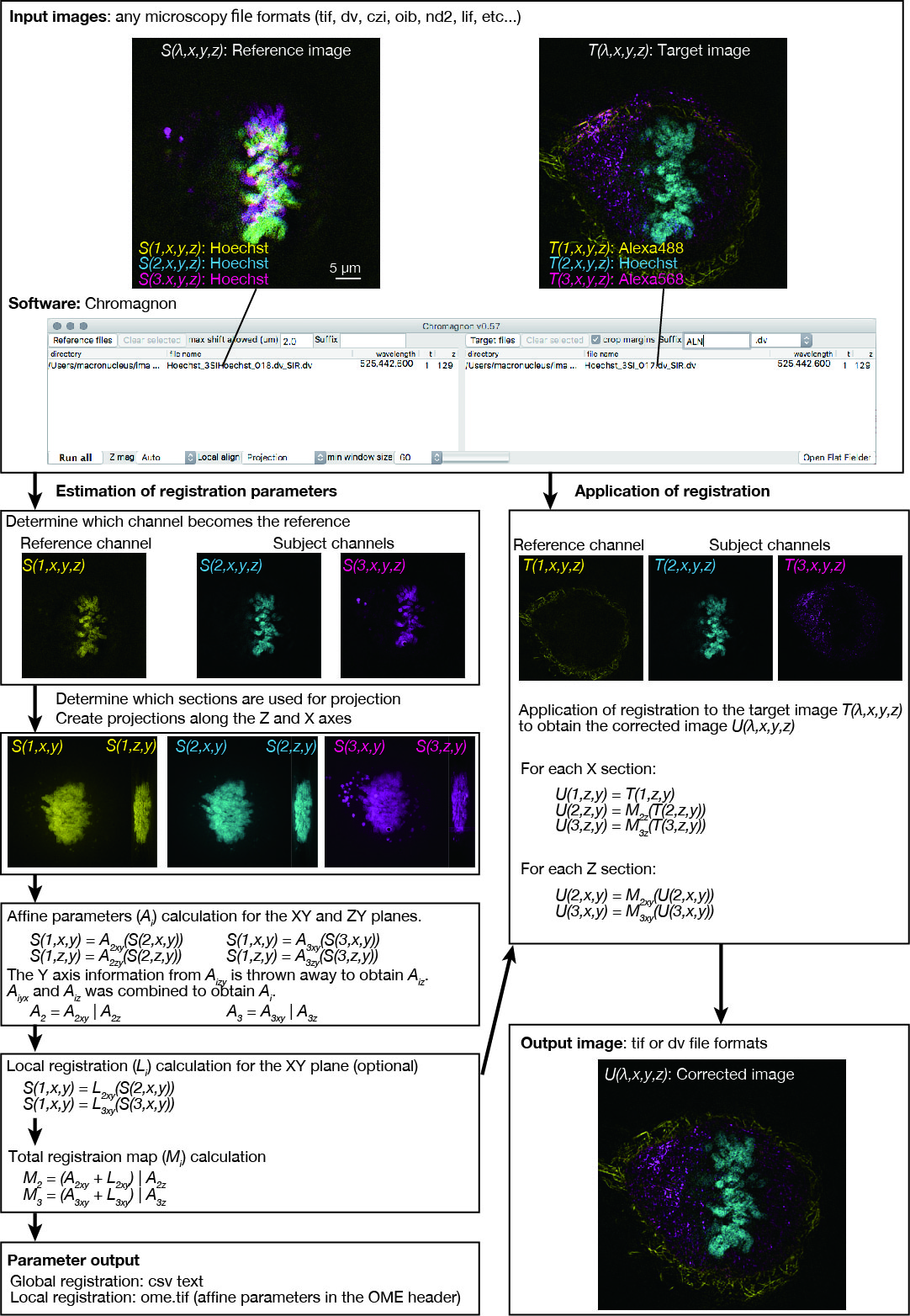


Scale bar indicates 5 µm for the larger panels and 10 µm for the smaller panels.

**Fig. S7. Chromatic shift measurements using bright field images.**


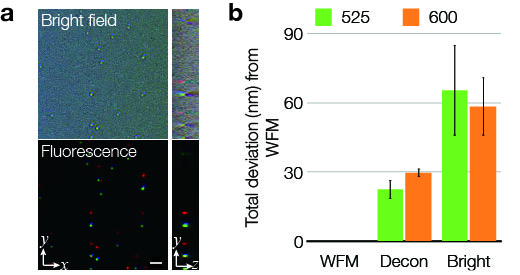


(**a**) Bright field and fluorescent WFM images of a single-layer multicolor bead slide were obtained in their respective color channels. Although bright field images are projections of transmission light, 3D stacks of such images contain vertical information as contrast reversal above and below the focus. The scale bar is 2 µm. (**b**) Global registration parameters were obtained from fluorescence WFM, deconvolved WFM, and bright-field images. Then, the relative differences to the seven global registration parameters obtained from WFM were calculated.

**Fig S8. Microscope setup used in this study.**


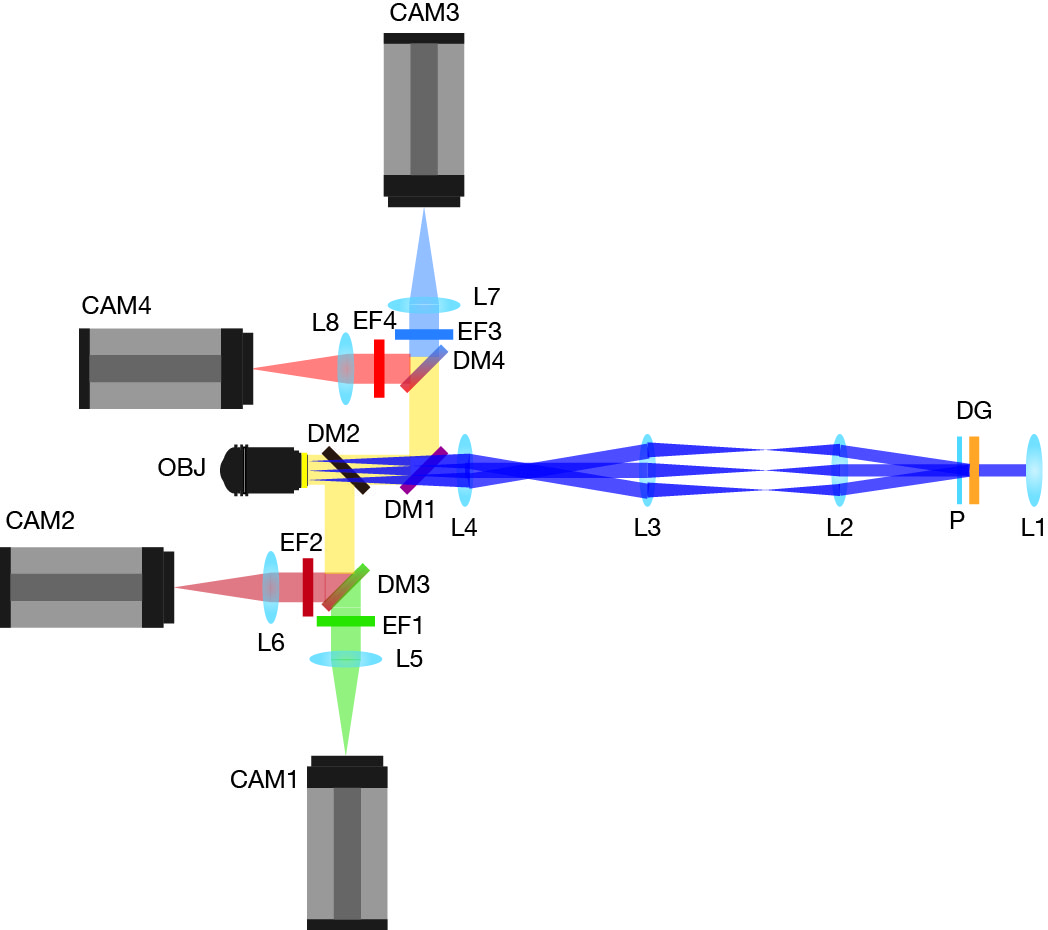


We used a DeltaVision OMX v3 with some modifications to use silicone immersion objective lens for 3D-SIM. Light from the multimode fiber (not shown) is collimated with lens (L1) and goes through a diffraction grating (DG) and a polarizer (P). The dispersed light is collected with L2, with a focal length of 70 mm instead of the default 75 mm for oil immersion objective lenses. After going through lens L3 and L4, the +1, 0, −1 order beams enter the pupil of the objective lens (OBJ) to create three-dimensional stripes necessary for 3D-SIM data acquisition. Fluorescence images were separated by dichroic mirrors (DM1–4), filtered with emission filters (EF1–4) and acquired with four cameras (CAM1–4), which can do simultaneous acquisition of up to four color channels. This microscope setup is efficient for 3D-SIM and the simultaneous measurement of multicolor images. Note, however, that nothing of this setup is essential for the proposed registration method itself.

**Fig. S9. Quality control for the local registration method.**


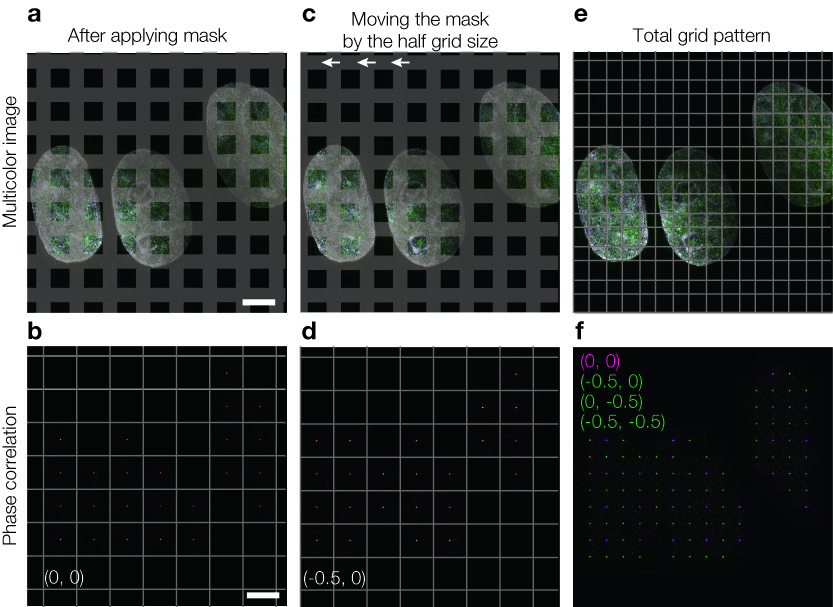


(**a**, **b**) In order to choose image regions with enough signal complexity, only the central quarter region (outside the grey lines) were examined to calculate variance. Scale bars are 5 µm. (**c**, **d**) To examine the missing regions (grey regions in **a**), the 7 × 7 grid was shifted by half the grid size (−0.5, 0) to examine different parts of the missing regions. (**e**, **f**) Repeating the shift in the X, Y, or both axes results in a 14 × 14 grid, and thus 14 × 14 peaks of phase correlation. (**f**) The peaks from the unshifted grid are shown in magenta and those from shifted grids are shown in green.

**Table S1. Example affine registration parameters.**

| **Fig.** | **Channel**  (nm) | **N_Z_** | **T_X_**  (µm) | **T_Y_** (µm) | **T_Z_** (µm) | **M_X_**  (times) | **M_Y_**  (times) | **M_Z_**  (times) | **R_Z_**  (degrees) |
| --- | --- | --- | --- | --- | --- | --- | --- | --- | --- |
| 1a | 600 | 101 | -0.150 | -0.185 | 0.467 | 0.9998 | 0.9994 | 0.9921 | -0.474 |
| 1c | 600 | 37 | 1.052 | -0.889 | -0.115 | 0.9889 | 0.9879 | 1.0540 | 0.006 |
| 4 | 600 | 49 | -0.245 | -0.326 | 0.090 | 0.9896 | 0.9890 | 0.9954 | -0.014 |
| 4 | 600 | 49 | -0.226 | -0.310 | 0.087 | 0.9895 | 0.9890 | 1.0044 | -0.013 |
| 4 | 600 | 49 | -0.221 | -0.306 | 0.093 | 0.9896 | 0.9888 | 1.0008 | -0.014 |
| 4 | 442 | 49 | -0.110 | -0.311 | -0.228 | 0.9850 | 0.9859 | 1.0036 | -0.488 |
| 4 | 442 | 49 | -0.104 | -0.287 | -0.210 | 0.9848 | 0.9858 | 1.0000 | -0.486 |
| 4 | 442 | 49 | -0.108 | -0.286 | -0.209 | 0.9851 | 0.9860 | 0.9968 | -0.483 |
| 6a | 623 (ex561) | 17 | 0.000 | 0.042 | 0.313 | 0.9905 | 0.9906 | 1.0000 | 0.0002 |
| 6a | 623 (ex561) | 21 | -0.004 | 0.036 | 0.354 | 0.9907 | 0.9906 | 1.0000 | 0.0000 |
| 6a | 623 (ex561) | 20 | -0.007 | 0.031 | 0.344 | 0.9904 | 0.9908 | 1.0000 | 0.0079 |
| 6a | 623 (ex488) | 17 | -0.001 | -0.001 | 0.004 | 0.9998 | 0.9998 | 1.0000 | -0.0014 |
| 6a | 623 (ex488) | 21 | -0.003 | -0.001 | 0.000 | 1.000 | 0.9999 | 1.0000 | -0.0081 |
| 6a | 623 (ex488) | 20 | -0.003 | -0.001 | 0.000 | 1.0001 | 0.9999 | 1.0000 | 0.0085 |
| 7b | 609 | 93 | -0.107 | -0.016 | 0.116 | 1.0002 | 0.9994 | 0.9986 | -0.4680 |
| 7b | 609 | 103 | -0.11 | -0.008 | 0.106 | 1.0001 | 0.9991 | 1.0001 | -0.4599 |
| 7b | 609 | 69 | -0.117 | -0.008 | 0.100 | 1.0002 | 0.9994 | 0.9927 | -0.4672 |

Registration calculation was performed with quadrisection phase correlation using green channel (525 nm) as a reference. **N_Z_** is the number of Z section. Data for different figures may be acquired with different camera positioning and objective lenses. The data in Fig. 6a was calculated without magnification along the Z axis due to small number of optical slices.

**Table S2 Registration methods and microscopy used in this study.**

| **Fig.** | **Reference for channel alignment** | **Microscopy** | **Registration** |
| --- | --- | --- | --- |
| 1a-b | Actin stained with Alexa Fluor 488 and 594 | 3D-SIM | Global |
| 1c-d | DNA stained with DAPI | 3D-SIM | Global |
| 1e-f | treacle and fibrillarin, stained with Alexa Fluor 488 and 568 | 3D-SIM | Global |
| 2 | Simulated images | WFM | Global |
| 3 | DNA stained with DAPI | 3D-SIM |  |
| 4 | 0.2 µm Multispectral beads | 3D-SIM | Global and local |
| 5a-b | 0.2 µm Multispectral beads | 3D-SIM | Global |
| 5c | 1.0 µm Blue beads | WFM | Global |
| 5f | 0.2 µm Multispectral beads and DAPI image | 3D-SIM and WFM | Global |
| 5e | DNA stained with DAPI | 3D-SIM | Global |
| 5f | 0.2 µm Multispectral beads and DAPI image | 3D-SIM | Global |
| 6b | Actin stained with Alexa Fluor 488 and 594 | CLSM | Global |
| 6d, 7 | Actin stained with Alexa Fluor 488 and 594 | 3D-SIM | Global |
| S1,2 | Simulated images | WFM | Global |
| S3 | DAPI and 0.2 µm Multispectral beads | 3D-SIM | Local |
| S4 | Simulated images | WFM | Local |
| S5 | 0.2 µm Multispectral beads | 3D-SIM | Global and local |
| S6 | DNA stained with Hoechst | 3D-SIM | Global and local |
| S7b | 0.2 µm Multispectral beads | WFM,  bright filed | Global |
| S9 | DNA stained with DAPI | 3D-SIM | Local |
